# Distribution patterns of soil bacteria, fungi, and protists emerge from distinct assembly processes across subcommunities

**DOI:** 10.1002/ece3.11672

**Published:** 2024-07-10

**Authors:** Alexis Kayiranga, Alain Isabwe, Haifeng Yao, Huayuan Shangguan, Justin Louis Kafana Coulibaly, Martin Breed, Xin Sun

**Affiliations:** ^1^ Key Laboratory of Urban Environment and Health, Ningbo Observation and Research Station, Institute of Urban Environment Chinese Academy of Sciences Xiamen China; ^2^ University of Chinese Academy of Sciences Beijing China; ^3^ Zhejiang Key Laboratory of Urban Environmental Processes and Pollution Control CAS Haixi Industrial Technology Innovation Center in Beilun Ningbo China; ^4^ College of Science and Engineering Flinders University Bedford Park South Australia Australia

**Keywords:** assembly processes, distribution patterns, soil microbial domains, urban soil

## Abstract

Environmental change exerts a profound effect on soil microbial domains—including bacteria, fungi, and protists—that each perform vital ecological processes. While these microbial domains are ubiquitous and extremely diverse, little is known about how they respond to environmental changes in urban soil ecosystems and what ecological processes shape them. Here we investigated the community assembly processes governing bacteria, fungi, and protists through the lens of four distinct subcommunities: abundant, conditionally rare, conditionally abundant, and rare taxa. We show that transient taxa, including the conditionally rare and conditionally rare or abundant taxa, were the predominant subcommunities. Deterministic processes (e.g., environmental filtering) had major roles in structuring all subcommunities of fungi, as well as conditionally rare and abundant protists. Stochastic processes had strong effects in structuring all subcommunities of bacteria (except rare taxa) and conditionally rare protists. Overall, our study underscores the importance of complementing the traditional taxonomy of microbial domains with the subcommunity approach when investigating microbial communities in urban soil ecosystems.

## Graphical abstract

We classified soil taxa from urban soil system into different subcommunities and studied the assembly of those subcommunities using the neutral community model.

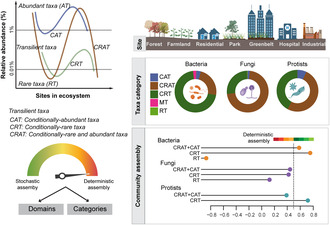

## 1 INTRODUCTION

Soil harbors the largest portion of the planet's biodiversity—more than half of all life forms (Anthony et al., 2023). The possibility of soil biodiversity extinction associated with land use changes and intensification jeopardize the sustainability of our planet (Ellis, 2021; Foley et al., 2005; Geisen et al., 2019; Veresoglou et al., 2015). There is a pressing need to evaluate variation in soil biotic compositions to inform evidence‐based policies and practices that aim to protect soil biodiversity (Sutherland et al., 2022).

Urbanization changes the properties and structure of the soil which in turn affect distribution patterns of soil microbial communities (Ellis, 2021; Sun et al., 2023; Yi et al., 2022). Soil microbial communities can potentially act as a sentinel to assess soil health in urban settings (Sun et al., 2023). How urban soil system—which typically spans a small geographic area with many environmental extremes—affect microbial communities is poorly represented in literature (Nugent & Allison, 2022). Until fairly recently, researchers have started to explore soil microbial diversity in relation with urbanization highlighting substantial differences in community compositions among urban edaphic patches, and more intense abiotic stress than natural environments (Delgado‐Baquerizo et al., 2021; Isabwe et al., 2022; Liao et al., 2022).

Multiple facets of soil microbial communities including classical taxonomic groups, the niche breadth‐based (an approach that categorizes microbial taxa based on their ability to occupy a wide range of ecological niches, and typically divides taxa into two main groups: generalists and specialists), and the abundance‐based classification have been foci for evaluating microbial responses to environmental changes in different ecosystems. Mejia et al. (2023) explored concurrently soil bacterial, eukaryotic, and fungal communities influenced by a long‐term contamination in urban brownfields. Banerjee et al. (2024) showed that biotic homogenization in arable farming potentially contributed to the transition of some fungal groups become fewer or absent in arable than droughts soil mycobiome across Europe. Von Meijenfeldt et al. (2023) suggested that, depending on their metabolism and habitat preferences, distribution patterns differ among bacteria specialist and generalist taxa. Xu et al. (2022) found that microbial generalists and specialists contributed differently to the total community diversity in farmland soils. Another facet arises from viewing microbial communities as composed of many rare species and a few abundant taxa (Bickel & Or, 2021; Jousset et al., 2017; Shade et al. 2014). These abundance‐based groups (hereafter, subcommunities) have also been instrumental to delineate mechanisms that shape microbial community patterns. For example, Delgado‐Baquerizo et al. (2018) narrowed down a global atlas of soil bacterial community to a relatively small list of the “most wanted” taxa. Bickel and Or (2021) highlighted a “chosen few” which are common species that shape bacterial communities across global biomes. Although this approach has advanced our understanding of microbial community assembly and resilience in soil systems, it has placed limited emphasis on transient taxa which are taxa whose abundance increase or decrease under certain conditions.

Some microbial community members can be active while others within this community remain dormant or inactive over extended periods (Campbell et al., 2011; Lennon & Jones, 2011; Logares et al., 2013). Uncommon microbial community members make up the rare taxa and represent a significant share of microbial diversity despite their low abundance. Rare taxa also have the capacity to contribute to a “seed bank” that maintains the stability of a microbial community due to their extensive diversity and rapid adaptability to changes in the environment (Logares et al., 2013; Nyirabuhoro et al., 2020). Abundant taxa, opposite to rare taxa, comprise a small number of distinct taxa that are present in high abundance (Pedrós‐Alió, 2012; Sogin et al., 2006). Compared to abundant or rare taxa, transient taxa exhibit higher temporal and/or spatial variation (Lee et al., 2021; Unzueta‐Martínez et al., 2022). These include the rare microbial taxa that can occasionally become very abundant (conditionally rare taxa (CRT); Shade et al., 2014) or the abundant taxa that occasionally become very rare (conditionally abundant taxa (CAT), Nyirabuhoro et al., 2020; Zhu et al., 2023). The third category of transient taxa includes the conditionally rare or abundant taxa (CRAT) which are taxa that fluctuate among both the abundant and the rare taxa (Nyirabuhoro et al., 2020). Despite these differences in abundance, little is known about the dynamics and mechanisms of these transient taxa in urban soil ecosystems.

Delineating ecological processes that shape soil microbial community composition and structure (i.e., community assembly) has been a longstanding and unanswered question in ecology. Deterministic and stochastic assembly processes are associated with the niche and neutral theories, respectively (Shi et al., 2018). Deterministic assembly emphasize the differences among species, inter‐species interactions and selective pressure by the local environment (He et al., 2021). In contrast, stochastic assembly assumes that equivalence and randomness structure community composition. It is important to note that both stochastic and deterministic processes act in a concert. Because of their unequal distributions across spatial and temporal axes, the relative importance of either process can hypothetically change for microbial subcommunities in soils (Wan et al., 2021; Yang et al., 2022). This may be partially explained by the fact that soil microbial communities are influenced by both biotic and abiotic attributes. On the one hand, soil properties such as pH and moisture may contribute to filter out some microbial community members (Philippot et al., 2023; Wang et al., 2024). On the other hand, biotic factors such as symbiosis, competition, trade‐offs, and predation influence how the different taxa interact in the soil (Wardle et al., 2004). Furthermore, rare taxa may enter a state of extended dormancy due to disturbances in the environment (Lennon & Jones, 2011). Philippot et al. (2021) showed that extended dormancy can increase immigration of species. According to Goss‐Souza et al. (2017), soil microbial community assembly depend largely on land use. Shi et al. (2018) and Yang et al. (2021) explained that microbial communities are structured by demographic occurrence and various dispersal modes in the environment. Studies conducted by Xue et al. (2018), Yang et al. (2021), and Yang et al. (2022) showed that abundant and rare taxa have significant different features and functional traits, highlighting the importance of rare taxa in maintaining ecosystem functions. For example, rare taxa may help reduce sulfate, break down organic matter, and resilience to environmental disturbances, in comparison to abundant taxa (Cao et al., 2020; Yang et al., 2021). Such functional attributes of microbial communities vary across microbial domains. Bacteria, fungi and protists in soils exhibit diverse functional roles, such as decomposition, nutrient cycling, and symbiotic associations with plants (Fierer et al., 2007; Sun et al., 2023), which can be harnessed in urban soil systems to improve soil health, enhance plant growth, and promote sustainable land management practices (Bender et al., 2016).

Even though several studies have been conducted to understand rare taxa, little is known about the community assembly process of CRAT, CAT, CRT, and rare taxa. Fortunately, the rapid advancement of molecular‐based approaches for microbial community profiling has made it easier to understand their distribution patterns in soils (Pajares et al., 2016; Wall et al., 2008). These methods have enabled scientists to explore a typical microbial community, contributing to the comprehension of the diversity and dynamic composition of bacterial, fungal, and protist communities at an unprecedented level. An important question is how similar abundant, rare, and transient taxa responses are to environmental changes. Recent studies have demonstrated that classifying microbial communities into subcommunities based on their abundance patterns can provide valuable insights into their ecological functions and responses to environmental changes (Jiao et al., 2017; Li et al., 2021; Singavarapu et al., 2023; Wang et al., 2023). To better understand urban soil microbial communities, it is essential to investigate their subcommunities which may be associated with various edaphic patches (i.e., distinct spatial units or areas within the urban landscape that exhibit unique soil characteristics) within urban ecosystems.

Here we aimed to explore the distribution patterns and community assembly processes that govern these soil microbial subcommunities in urban soils. First, we investigated whether there were comparable responses of abundant taxa to environmental changes across three domains of microbial populations—bacteria, fungi, and protists. Second, we examined whether there were stronger responses of transient taxa (i.e., CRT, CAT, and CRAT) to specific aspects of environmental variation in urban soils, which vary along an environmental gradient. These variations encompass factors such as pH levels, moisture content (MC), total phosphorus (TP), total carbon (TC), total nitrogen (TN), and carbon–nitrogen (CN) ratio. We aimed to understand how these factors influence the responses of transient taxa in urban soil habitats. Finally, we hypothesized that the relative contributions of deterministic and stochastic processes would vary depending on subcommunities rather than taxa domains because subcommunities (e.g., rare vs. abundant) can exhibit different responses to environmental changes (Bickel & Or, 2021; Delgado‐Baquerizo et al., 2018; Philippot et al., 2021).

## 2 MATERIALS AND METHODS

### 2.1 Habitat and site selection

**FIGURE 1:**
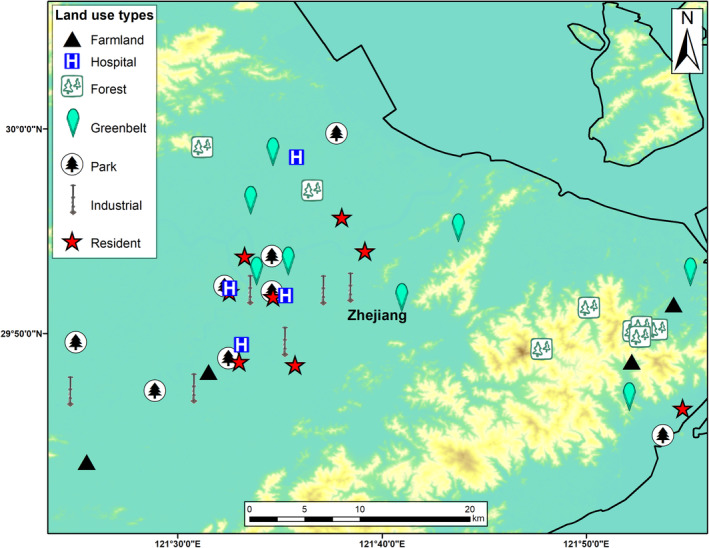
The geographic location of the sampling sites in urban ecosystems in Ningbo, China.

Sampling campaigns were carried out in Ningbo City (China) in June 2021 at 46 sites spanning seven different land use types. These land use types include farmland, forest, greenbelt, hospital, industrial, park, and residential (Figure 1). Our sampling design consisted of eight replicate sampling points at four land use types (forest, greenbelt, park, and residential), and four replicates at two land use types (farmland and hospital outdoor soil), and soils in industrial area which had six replicated sampling points. At each site, we randomly selected a 20 m × 20 m plot and within that plot, we extracted nine soil cores using a 5.5‐cm diameter auger. These cores were taken from a depth of 0–10 cm. Collected cores were homogenized and combined to create a composite sample. Six environmental variables (i.e., soil MC, pH, TC, CN ratio, TN, and TP) were measured following established protocols (Wall et al., 2008). In addition to these measurements, geographic coordinates of sampling sites were recorded for further analyses. The rationale for sampling soil samples in seven urban edaphic patches was to maximize our effort in detecting the most representative soil microbial taxa, including the rare and dominant, in an urban setting given the potential for local adaptation (Isabwe et al., 2022).

### 2.2 DNA extraction, sequencing, and bioinformatics

Total soil DNA was extracted from a 0.5‐g homogenized soil sample using the FastDNA® Spin Kit for soil (MP Biomedicals, Santa Ana, CA, USA), following the manufacturer's protocol. The extracted total soil DNA was eluted in a 100 L DES and spectrophotometric methods via the NanoDrop ND‐2000 (Thermo Fisher Scientific, Wilmington, USA) were used to assess the quantity and quality of the extracted DNA. High throughput sequencing targeting the V4 region of the eukaryotic 18S rRNA gene, the V4‐V5 region of the 16S rRNA gene, and the fungal internal transcribed spacer 1 (ITS1) region, respectively, was done to analyze the eukaryotic (i.e., protists), bacterial and fungal communities in soils (Fadeev et al., 2021). The 18S rRNA gene and 16S rRNA gene PCR reactions were conducted in three parallels in 20.0 μL mixtures consisting of 0.4 μL Trans Start® FastPfu DNA Polymerase (Transgen Biotech Co., Ltd., Beijing, China), 4.0 μL of 5× TransStart® FastPfu Buffer, 2.0 μL of 2.5 mM dNTPs, 0.8 μL of each primer (5 μM), 0.2 μL BSA (2 mg·mL^−1^), 2 μL of template DNA (10 ng·μL^−1^), and 9.8 μL of sterilized ddH_2_O. The ITS1 region was amplified in a total volume of 20 μL in triplicate, the PCR mix contained 0.2 μL Premix Taq™ DNA Polymerase (Takara Biotechnology, Dalian, China), 4.0 μL of 10× Buffer, 2.0 μL of 2.5 mM dNTPs, 0.8 μL of each primer (5 μM), 0.2 μL BSA (2 mg·mL^−1^), 2 μL of template DNA (10 ng·μL^−1^), and 11.6 μL of sterilized ddH_2_O. Negative control samples were also included throughout the PCR assay to ensure reaction systems were not contaminated. The PCR assays were performed using an ABI Geneamp® PCR System 9700 thermocycler (Applied Biosystems, San Diego, CA, USA). The PCR products were recovered from a 2% agarose gel and purified using the AxyPrep DNA Gel Extraction Kit (Axygen Biosciences, Union City, CA, USA) according to the manufacturer's protocols and quantified using Quantus™ Fluorometer (Promega, USA) (Fan et al., 2019), and then sequenced on the Illumina MiSeq® PE 300 platform (Majorbio Bio‐Pharm Technology Co. Ltd., Shanghai, China).

The FLASH 1.2.11 program was used to align the pair‐end reads and q‐score plugin. Low‐quality reads with quality scores below 20 were excluded by means of FASTP 0.19 software. The operational taxonomic units (OTUs) were clustered by sequence similarity, with a 97% cutoff. Chimeric sequences were removed from the data set using UPARSE 7.0 (Edgar et al., 2011). One nucleotide was selected randomly from each OTU and used for taxonomic identification. Using RDP 2.11 c Classifier, the taxonomic status of OTUs was determined. In this study, we refer to protists as a taxonomic group encompassing all eukaryotic microorganisms excluding metazoa. This definition is consistent with the scope of our analysis, which focuses on characterizing soil microbial communities across bacteria, fungi, and protists. By excluding metazoa, we aim to specifically investigate the diversity and distribution patterns of non‐animal eukaryotic organisms within the soil ecosystem. We used the Protist Ribosomal Reference (PR2; Quast et al., 2013), SILVA rRNA database v138, and UNITE fungal ITS database v8.0 (Nilsson et al., 2019) to assign taxonomic classifications to soil eukaryotes, bacteria, and fungi, respectively. To examine protist taxa exclusively, we removed all taxa that were classified as metazoa from the eukaryote dataset. All reads related to archaea and chloroplasts were removed from the bacterial community before the final OTU table was generated.

### 2.3 Defining of the subcommunities

The original dataset for bacteria, fungi, and protists consisted of 9908, 9902, and 8117 OTUs, respectively. Before classifying taxa based on their relative abundances (conditionally rare or abundant, rare, and CRT), all samples were rarefied to maintain an equal number of sequences across the samples (Nyirabuhoro et al., 2020). The rarefaction curves generated in the R software (Figure S1 and Table S1) indicated that the sequencing effort was sufficient to capture the majority of the microbial diversity present in each sample. This approach ensures that our analysis is not biased towards samples with higher sequencing depth, thus providing a more accurate representation of the true microbial community composition.

As previously described in the literature, a more comprehensive description of the criteria was used to define and categorize the four microbial subcommunities as follows. The classical cutoff level which designates rare OTUs (0.1 or 0.01%) and abundant OTUs (1%) was used to define abundant and rare taxa (Xue et al., 2018). These two categories may overlook the oscillating and intermediate taxa (those with a relative abundance between 0.1 or 0.01 and 10%, i.e., rare and abundant under certain conditions). In this study, we divided all OTUs into four subcommunities as follows: CAT, with a relative abundance ranging from rare (0.01%) to abundant (1%) in some samples (Chen et al., 2017; Dai et al., 2017); rare taxa, with a relative abundance 0.01% in all samples; CRT, with a relative abundance 0.01% in some samples but never 1% in any sample; and conditionally rare and abundant taxa. Taxa were divided into three sub‐communities: those that were present in large numbers, those that only occurred under certain conditions (perhaps owing to random factors), and finally, those at the extreme ends of the abundance spectrum, including both the exceptionally rare and the exceptionally abundant taxa, which were grouped together. The rare taxa (RT) and CRT were treated as separate groups, but CAT and CRAT were grouped together (Chen et al., 2019). The delineation of our subcommunities was driven by a need to capture the diverse abundance patterns within the total microbial community and provides insights into the distribution patterns and ecological roles of different taxa, enhancing our understanding of microbial community assembly processes.

### 2.4 Statistical analyses

Statistical analyses and data visualizations were done using R 4.0.3. This vegan package allows us to examine the distribution patterns of rare, abundant and transient taxa. For instance, we used analysis of similarity (ANOSIM) to see whether there were differences in domains between subcommunities (Nyirabuhoro et al., 2021). The data visualization‐related R packages used were *ggplot2* and *ggpubr* packages (Kassambara, 2023; Wickham et al., 2016). A pairwise environmental distance between sites was regressed against Bray–Curtis similarity matrices. Their relationships were assessed via Mantel tests, and their significance levels was estimated through the 999 permutations using *Ecodist* package (Goslee & Urban, 2022). To calculate environmental distance, we compiled a multidimensional matrix of environmental variables measured at each sampling site. These environmental variables encompassed a range of abiotic factors known to influence microbial community composition, such as soil pH, MC, temperature, and nutrient levels. Next, we employed standardization techniques to ensure that all environmental variables were on comparable scales and weighted equally in the calculation of environmental distance. This step is crucial for mitigating biases introduced by differences in the measurement units and magnitudes of individual environmental variables. Subsequently, we computed pairwise similarities between sampling sites based on the multidimensional environmental matrix using appropriate distance metrics, such as Euclidean distance or Bray–Curtis similarity. These distance metrics capture the overall similarity in environmental conditions between sites, accounting for both the magnitude and direction of differences across environmental variables. A Variation partitioning analysis was carried out using the *picante* package with an aim to examine the relative importance of environmental variables and spatial variables in explaining changes in soil microbial subcommunities. Spatial variables were the principal coordinates of neighboring matrices (PCNM). Forward‐selection was simultaneously performed on environmental and spatial variables to select the important environmental and spatial factors that had a great influence on subcommunities. Furthermore, we used a redundancy analysis via the vegan package to determine the most influential environmental drivers of microbial taxonomic assembly.

The neutral community model was used to evaluate the potential significance of stochastic process shaping the studied subcommunities of bacteria, fungi, and protists (Sloan et al., 2006). Stochastic processes are differentiated from deterministic processes based on their random nature and lack of predictability. This model describes patterns in the three domains in terms of scale. In our work, the criteria used to differentiate stochastic and deterministic processes include the probability of movement, compositional drift, and extinction probabilities that influence the structure of bacteria, fungi, and protists communities. The strength of stochastic processes in the model was assessed by the percentage of model fit, represented by *R* ^2^. *N* is the size of a metacommunity and involves migration, with its rate measured by the parameter Nm. The value is determined by multiplying the metacommunity size (that is, the total number of species in the whole region) with a subcommunity dispersal rate (Nyirabuhoro et al., 2020; Sloan et al., 2006). The neutral community model was run using *Hmisc*, *minpack.lm*, and *stats4* packages.

## 3 RESULTS

### 3.1 Abundance‐based subcommunities of soil bacteria, fungi, and protists

**FIGURE 2:**
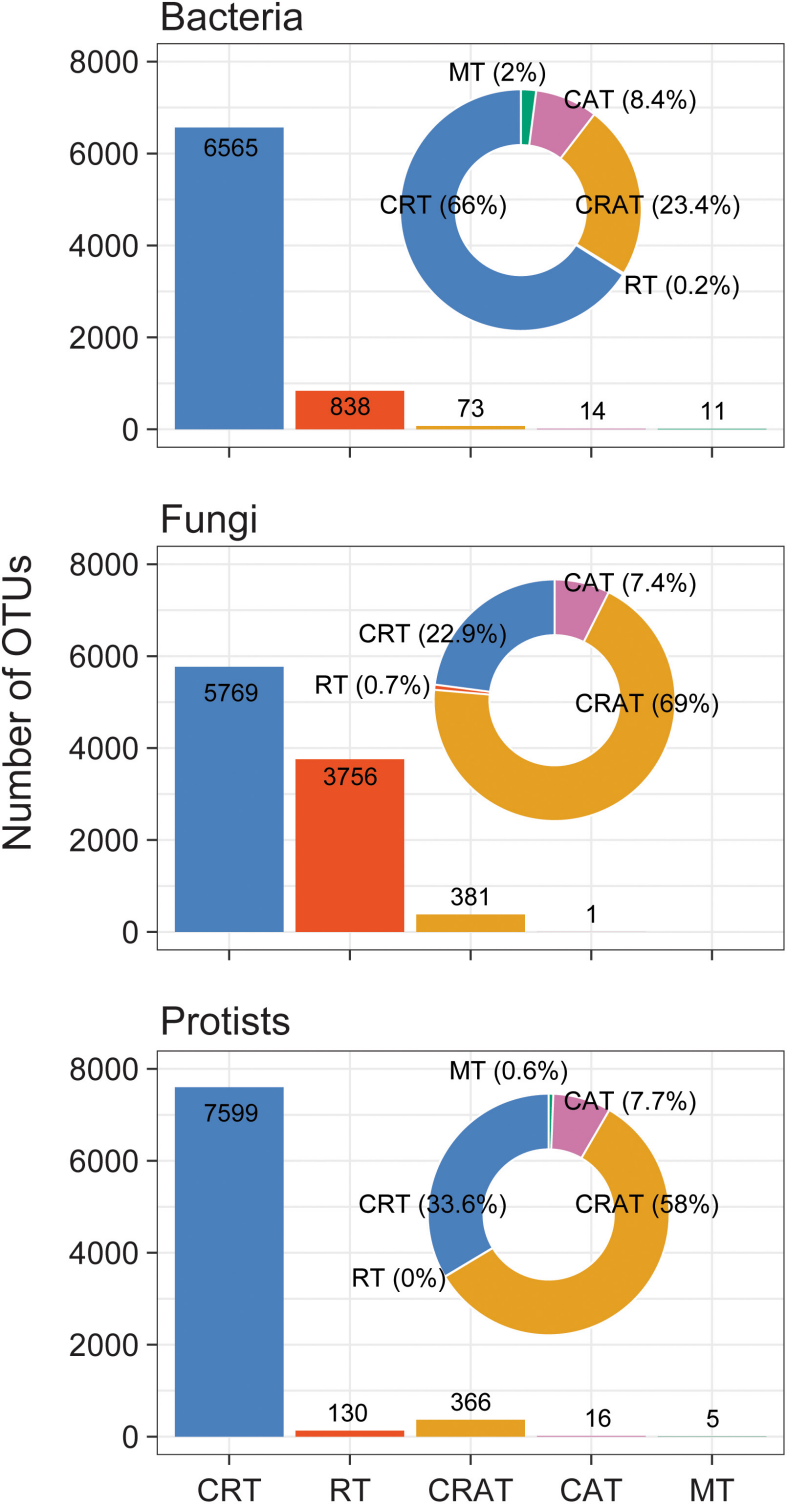
The relative number of OTUs (bar plots) and reads (pie charts) attributed to different sub‐communities. CAT, conditionally abundant taxa; CRAT, conditionally rare and abundant taxa; CRT, conditionally rare taxa; MT, moderate taxa; RT, rare taxa.

Bacterial (*n* = 728,791 reads), fungal (*n* = 1,658,393 reads), and protistan (*n* = 355,350 reads) communities were standardized by rarefaction of OTUs to allow comparison across samples. The original tables of OTUs for bacteria, fungi, and protists were rarefied to 7501, 9907, and 8116 OTUs, respectively. Rarefied tables for each microbial domain were then classified into different taxa subcommunities as follows: for bacteria, CRT accounted for 66% of the total community, CRAT (23.4%), moderate taxa (2%), CAT (8.4%), and rare taxa (0.2%). For fungi, CRT were 22.9% of the total community, and CRAT (69%), CAT (7.4%), and rare taxa (0.7%) were defined. For protists, CRT were 33.6%, and CRAT (58%), CAT (7.7%), and moderate taxa (0.9%) were obtained from all reads (Figure 2). Moderate taxa refer to microbial taxa that exhibit a moderate level of abundance within a given community. These taxa are neither extremely rare nor overwhelmingly abundant compared to other taxa present. In the context of our study, moderate taxa may represent a middle ground in terms of their prevalence and impact on the overall microbial community composition. The relative abundance of moderate taxa in fungi was lower compared to bacteria and protists, which may have contributed to the absence of clear patterns in the fungi dataset. The dominant taxa in terms of both the number of OTUs and the number of reads of the studied subcommunities were the CRT and CRAT (Figure 2).

The most abundant bacterial phyla were Proteobacteria, Chloroflexi, and Planctomycetota. Proteobacteria in the three categories of CRAT, CAT, and CRT were significantly more abundant than bacterial in the rare category (Figure S2A). However, no clear patterns were observed on the phyla Acidobacteria, Actinobacteria, Bacteroidota, Firmicutes, Gemmatimonadota, Myxococcota, and Planctomycetota in all subcommunities. In addition, the most dominant fungal and protistan phyla in all subcommunities were Ascomycota and Stramenopiles, respectively (Figure S2B,C). Ascomycota and Stramenopiles showed greater dominance in CRAT, CAT, and CRT compared to rare taxa.

**FIGURE 3:**
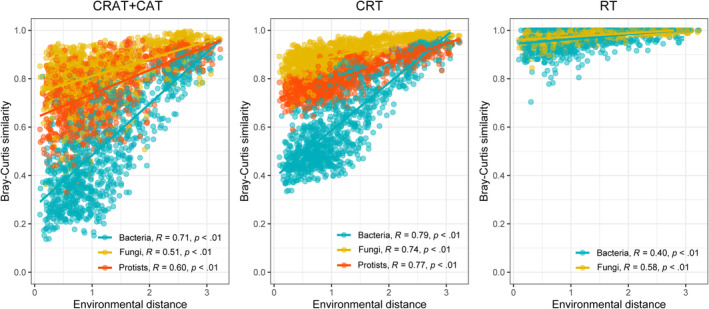
Correlations between community similarity and environmental distance. The plots are separated by the observed subcommunities for three domains (bacteria, fungi, and protists). The r indicates Mantel's correlation coefficient, based on the Spearman rank correlation. CAT, conditionally abundant taxa; CRAT, conditionally rare or abundant taxa; CRT, conditionally rare taxa; RT, rare taxa.

By correlating the environmental distance to community similarities (Figure 3), we observed coherent patterns among subcommunities. Only slight differences were observed for CRT which exhibited stronger correlations for bacteria (*r* = 0.79; *p* < .001) than for fungi (*r* = 0.74, *p* < .001) and protists (*r* = 0.77, *p* < .001). The correlation coefficients between community similarity matrices and environmental distance for the CAT and CRAT subcommunity were also slightly higher for bacteria (*r* = 0.71, *p* < .001) than that for protists (*r* = 0.60, *p* < .001) and fungi (*r* = 0.51, *p* < .001). Rare bacteria taxa had a weaker correlation coefficient (*r* = 0.40, *p* < .001) compared to rare fungi (*r* = 0.58, *p* < .001; Figure 3).

### 3.2 Environmental and spatial factors controlling subcommunities

**FIGURE 4:**
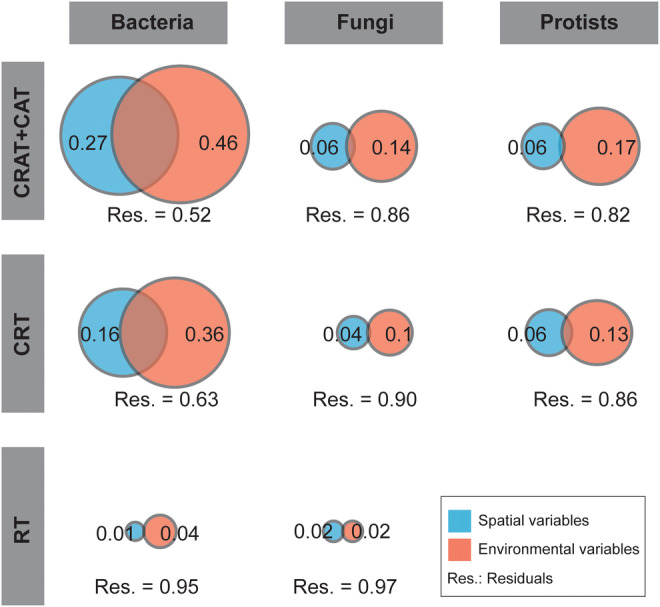
Results of the variation partitioning analysis. Only the percentage variance explained by environmental variables (moisture, pH, total phosphorus, total carbon, total nitrogen, and carbon–nitrogen ratio) and spatial variables (longitude and latitude) and the residual variance are shown. The remaining fractions are shown in Table S2. CAT, conditionally abundant taxa; CRAT, conditionally rare or abundant taxa; CRT, conditionally rare taxa; RT, rare taxa.

A substantial variance in community composition was explained by environmental variables in the three microbial domains than spatial variables, especially for the CRAT‐conditionally‐abundant taxa subcommunity as per variation partitioning analysis (Figure 4). This was observed for bacteria, protists, and fungi, where 46%, 14%, and 17% of community variation, respectively, was accounted for by environmental variables. While spatial variables explained the highest variance (16%) in the CRT subcommunity of the bacterial domain, their explanatory power was weaker than environmental variables in this subcommunity (36%). The same pattern was observed for fungi and protists, where environmental variables accounted for a larger share of the variation (Fungi: 10% and Protist: 13%) than spatial variables (Fungi: 4% and Protist: 6%). For rare taxa, both environmental and spatial variables explained <10% of community composition variation across all three domains. A considerable portion of community variation remained unexplained for the rare taxa, which was not present for other subcommunities for each microbial domain (Figure 4).

To determine environmental and spatial factors that significantly impacted bacterial, fungal, and protistan across subcommunities, we used a forward‐selection approach for variable selection (Table S2). This approach involves iteratively adding variables to the model based on their significance in explaining the observed patterns, allowing to identify the subset of environmental and spatial factors that collectively contributed the most to subcommunities of interest. Specifically, we found that CRAT and CAT of bacteria were primarily controlled by pH and CN ratio. CRT of both bacteria and protists were structured by pH, TP, CN ratio, and MC. For bacteria‐rare taxa, and protists (CRAT and CAT), TP, pH, and CN ratio were the most influential environmental factors. Additionally, pH, TP, and TC as the most important environmental factors for fungal‐CRAT and CAT. For rare fungal taxa, pH was the primary driving factor. TC primarily controlled rare bacterial subcommunities. Additionally, the PCNM that capture the main spatial patterns, were forward selected for each subcommunities (Table S2). For fungi‐CRAT‐CAT, the selected PCNM vectors included PCNM1, PCNM5, and PCNM20. For bacteria‐CRAT‐CAT, the selected PCNM vectors comprised PCNM1, PCNM20, PCNM7, and PCNM13. The specific PCNM vectors varied for different groups of organisms as shown in Table S2.

### 3.3 Community assembly processes of different subcommunities

**FIGURE 5:**
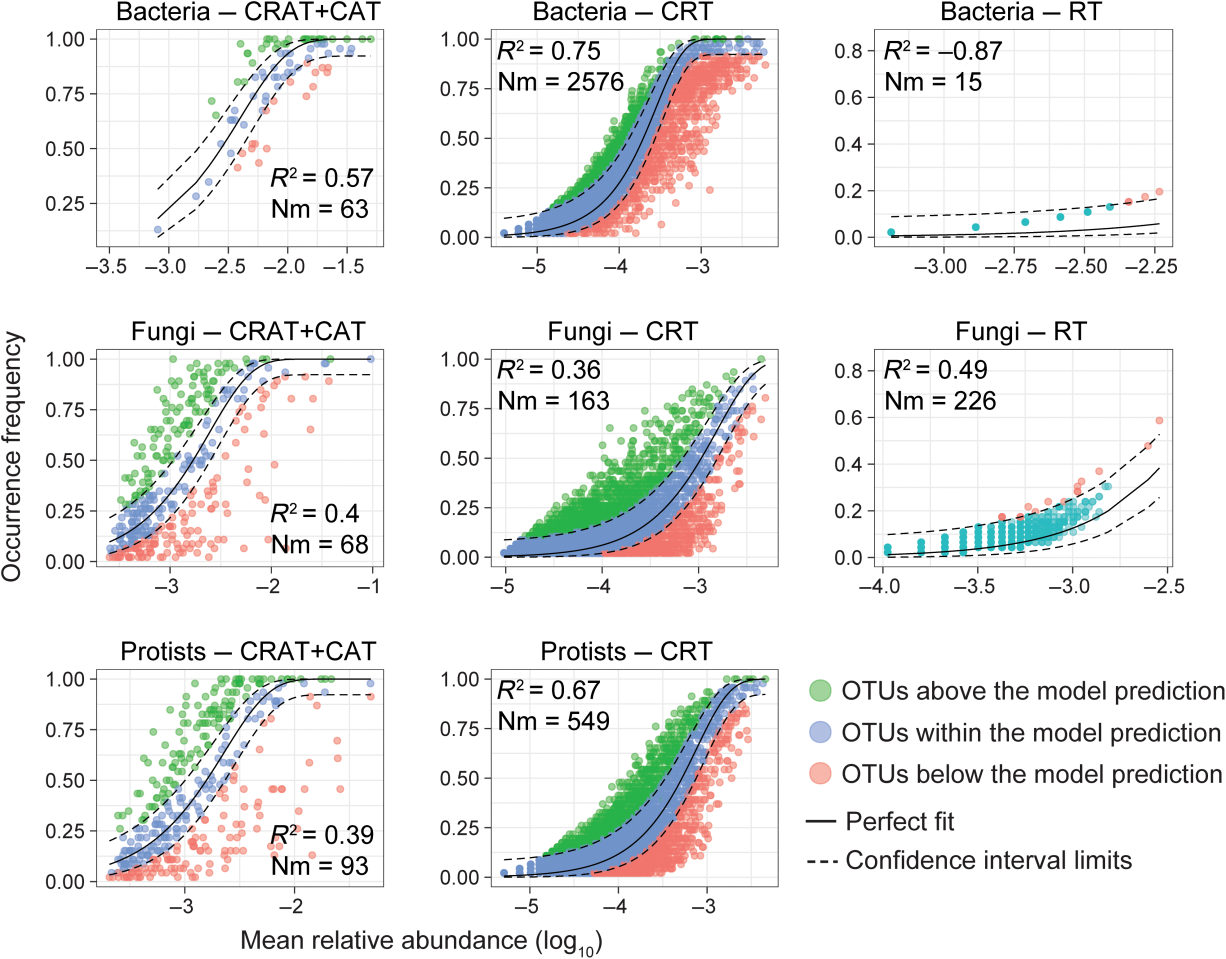
Neutral community model (NCM) analysis of bacteria, fungi, and protists (a–h) domains. The solid lines exhibit the best fit to the neutral community model (NCM), and the dashed blue lines represent 95% confidence intervals around the model prediction. *R* ^2^ and metacommunity size multiplied by immigration (*N*_m_) values indicate the model's goodness of fit.

The relative importance of stochastic processes structuring each subcommunity of the studied microbial domains was assessed using the neutral community model (Figure 5). The neutral model fit of CRT was higher (*R* ^2^ = 0.75 and *N*_m_ = 2576) than that of CRAT combined with CAT (*R* ^2^ = 0.57) for bacteria. For rare taxa, the *R* ^2^ value was negative (*R* ^2^ = −0.87) (Figure 5). For fungi, the neutral model of *R* ^2^ = 0.4 was found for the CRAT‐CAT subcommunity. This fit was slightly greater that of to the CRT (*R* ^2^ = 0.36) but stronger than that of the rare taxa (*R* ^2^ = 0.49) (Figure 5). Meanwhile, for protists, the CRT exhibited a better fit to the neutral model compared to the CRAT‐CAT whose neutral model fits were *R* ^2^ = 0.67 and *R* ^2^ = 0.39, respectively (Figure 5).

### 3.4 Effect of environmental variables on community composition of microbial subcommunities

**FIGURE 6:**
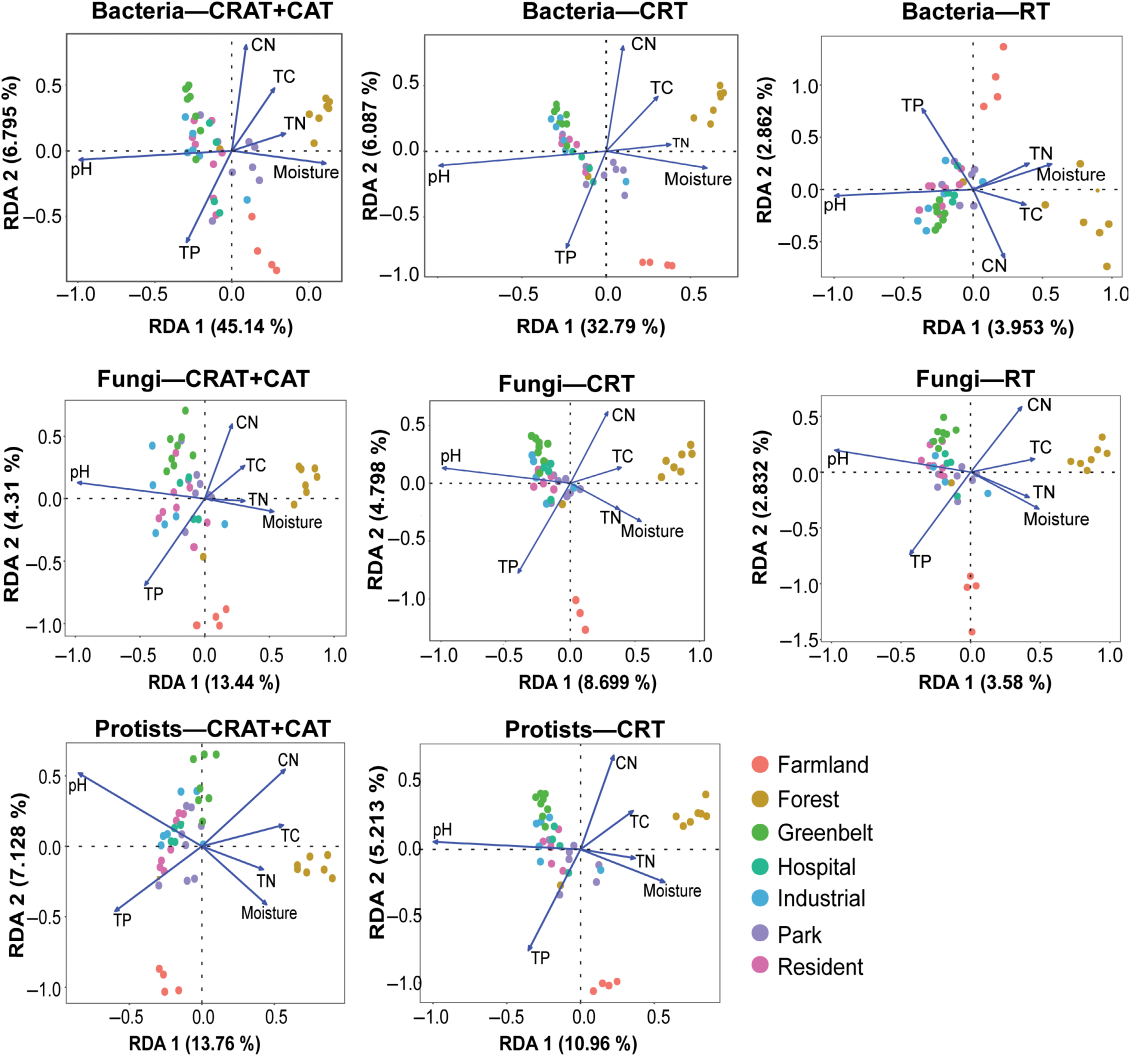
Redundancy analysis showing trends in significant environment variables and different subcommunities. CRAT‐CAT, conditionally rare or abundant taxa‐conditionally abundant taxa; CRT, conditionally rare taxa; RT, rare taxa.

By using redundancy analysis, we opted to determine environmental factors that have the strongest influence on community composition of the studied subcommunities and to which ecosystem type environmental factors tend to prevail. The variation in bacterial‐CRAT, CAT, and CRT composition was related to three environmental variables: TC, CN ratio, and TN which mainly prevail in forest ecosystems. There were two parameters (TN and moisture) that were correlated with the bacteria‐rare taxa composition prevailing potentially in farmland ecosystems. The subcommunities of fungi and protists were both frequently correlated with the four environmental variables namely CN ratio, TC, TN, and moisture (Figure 6).

## 4 DISCUSSION

To understand the differences among subcommunities, we examined the community compositions of bacteria, fungi, and protists sampled across seven different land use types. Our idea is in line with the understanding of subcommunities, as demonstrated by Singavarapu et al. (2023), who investigated the potential of soil microbial communities and their constituting subcommunities and taxa for their role in nutrient cycling. This research offers a critical mechanistic understanding for better management of forest soil ecosystems. Rather than focusing on traditional taxonomic classifications, we explored the concept of subcommunities to better understand microbial community functions and ecological effects. To this end, we determined whether CRT (transient taxa) follow a spatially explicit environmental gradient and explored the ecological processes driving these microbial subcommunities. Our findings suggest that CRT (transient taxa) play a crucial role in contributing to ecosystem stability and maintaining function, even in the presence of strong environmental fluctuations. These taxa have the ability to shift from a dormant to an active state, allowing them to adapt to changing conditions (Jiao & Lu, 2020; Nyirabuhoro et al., 2020, 2021; Wang et al., 2020).

Of the four studied subcommunities, the CRT emerged as the most dominant subcommunity. This highlights the importance of considering abundance‐based subcommunities rather than relying solely on classical taxonomic groups. Additional evidence that classifying microbial communities into subcommunities based on their abundance patterns can enhance our understanding of their ecological functions and responses to environmental factors has been portrayed in recent studies. Singavarapu et al. (2023) found that the functional potential of soil microbial communities and their subcommunities varied with tree mycorrhizal type and tree diversity, suggesting that subcommunity analysis can reveal important links between microbial diversity and ecosystem functions. Similarly, Li et al. (2021) demonstrated that abundant and rare subcommunities in paddy soil exhibited distinct patterns during wetting‐drying cycles, indicating that subcommunities may respond differently to environmental fluctuations and contribute to ecosystem stability in unique ways. Moreover, Jiao et al. (2017) showed that rare and abundant bacteria in oil‐contaminated soils had different biogeographic patterns and ecological diversity, highlighting the importance of considering subcommunities when investigating microbial community assembly processes and functions. Finally, Wang et al. (2023) found that rare and abundant microorganisms in soil responded differently to recurring biotic disturbances, further emphasizing the need to consider subcommunities when studying microbial community dynamics and resilience. Taken together, these studies provide additional evidence for the idea that classifying microbial communities into subcommunities based on their abundance patterns can reveal important insights into their ecological functions, responses to environmental factors, and roles in ecosystem processes. In addition, we focused on exploring the correlations between community similarity, environmental distance, and the use of the neutral community model in the domains of bacteria, fungi, and protists. These analyses provided an understanding of microbial community dynamics and assembly processes within the studied ecosystems. This understanding can have an indirect relation to our knowledge of microbial changes because environmental conditions play a key part in the activation and growth of microorganisms (Goss‐Souza et al., 2017; Philippot et al., 2021; Shade et al., 2014).

### 4.1 Significant distance–decay relationships and contribution of subcommunities in domain variation

In terms of abundance of different taxa or group of organisms within a particular ecosystem, DNA (total) analysis revealed that CRT subcommunities exhibited dominance across all microbial domains. This suggests that approximately half of the bacteria, fungi, and protists in our study sites transitioned between abundant and rare taxa in the DNA‐based community profiles (Lennon & Jones, 2011; Shade et al., 2014). While our study focused on DNA analysis, it is important to note that RNA (active) analysis would provide insights into the functional activity of these taxa. Previous studies have suggested that rare taxa, considering their potential to become dominant under favorable conditions, can be considered as “seed banks” within microbial communities (Kunin et al., 2010; Newton & Shade, 2016). Furthermore, it has been demonstrated that rare taxa transitioning from being uncommon in some samples to becoming abundant in others can potentially exhibit higher activity levels. It is important to acknowledge that our amplicon‐based approach does not provide insights into community functions, highlighting the need for future studies to address this gap using metagenomic and meta transcriptomic data (Shade & Handelsman, 2012; Shade et al., 2014). Analysis of mean relative abundance values based on the proportional frequencies of OTUs and reads revealed that CRT and CRAT were highly represented in the community structure compared with common taxa. In contrast, moderate and rare taxa, found in a small number of samples, were overshadowed by the widespread distribution of CRAT across sampling sites. Our findings align with a classical study by Hansk (1982), suggesting that core species can adapt more easily to environmental variables than rare species, occupying a smaller proportion of sample sites than species with large local population (Shade & Handelsman, 2012; Shade et al., 2014). For example, we found that in all domains, the taxa which are rare under certain conditions and taxa which are either rare or abundant under certain conditions had a marked level of great dominance in the number of OTUs and the number of reads, respectively. Other evidence indicates that the level of conditional rarity within a community is more closely linked to an environmental Euclidean distance compared with other sub‐communities. The study finds a strong relationship between pairwise environmental distance and community similarity for fungi and protists. This correlation suggests that these organisms tend to be found in closer surroundings. This correlation is consistent with previous studies by Aas et al. (2019) and Zhang et al. (2018).

Additionally, changes in the environment may produce species assemblages specifically suited to a particular site. These changes can influence the similarity of communities and form unique community structure (Hanson et al., 2012). Thus, changes in the structure of microbial communities and their effects on community similarity are mainly attributed to rare taxa (e.g., CRT) (Lennon & Jones, 2011). This might be caused by an aspect of their propensity to competition, commensalism, predation, and other ecological relationships (Jiao et al., 2017; Jiao & Lu, 2020). These findings suggest that CRT may have used as potential drivers in biogeochemical processes within urban soils (Lynch & Neufeld, 2015).

### 4.2 Major and minor effects of environmental factors on the microbial domains

The soil microbial community was directly affected by changes in land use. According to Goss‐Souza et al. (2017), its structure and composition were altered along with decreased diversity. As microbes play a key role in urban environments, they break down plant litter and facilitating the turnover of soil organic matter (Pajares et al., 2016). Conventionally, it is believed that bacteria are traditionally thought to play a significant role in urban ecosystems; besides the environmental and ecological processes of the environment being sustained by them, they are also important for the transformation and cycling of nutrients. The relationships between microbial communities and their environment, as well as variables that determine biodiversity, may both be enhanced by elucidating the patterns of dominant phyla (Egidi et al., 2019; Kramer et al., 2016). Soil‐inhabiting bacteria, fungi, and protists are ecologically significant groups of living organisms and require the identification of such dominance patterns. Soil inhabiting bacteria, fungi, and protists are essential groups that require the identification of dominance patterns. By describing the dominant ecological phyla of the bacteria, fungi, and protists found in urban soil samples collected from Ningbo City, we aimed to address this knowledge gap and compared our findings with previous research (Egidi et al., 2019). Overall, the analysis of the community composition revealed that the most dominant phyla through the three subcommunities (CRAT‐CAT, CRT, and rare taxa) included bacteria (Proteobacteria, Chloroflexi, and Planctomycetota), fungi (Ascomycota, Basidiomycota, and Rozellomycota), and protists (Cercozoa, Lobosa, and Stramenopiles).

The ratio of Proteobacteria to the total bacterial community composition did not show clear variation from forest to industrial soil samples. However, we observed an increasing trend in this ratio from greenbelt to hospital soil samples. Our results contrast with the results by (Goss‐Souza et al., 2017), where the relative abundance of Proteobacteria increased from forest to grassland soil. Proteobacteria can play a significant role in global carbon and nitrogen cycling, particularly in forest soils (Mendes et al., 2015). The primary soil environmental factors (pH and moisture distribution) affecting soil bacteria during the transition from a forest to a hospital, provided a clear explanation for these changes. The relative abundance of Ascomycota in the fungal domain was highest in CRT subcommunities, followed by CRAT‐CAT, but lowest in rare taxa along soil habitats. Being the largest fungal phylum, Ascomycota has a variety of enzymes that can break down recalcitrant substrates like cellulose, keratin, and lignin, making it essential for nutrient cycling in soil ecosystems (Bertini & Azevedo, 2022; Ko et al., 2017). Basidiomycota, the second‐largest phylum of fungi in our study, is well known for producing spores on basidia during sexual reproduction and for growing mushrooms (Ko et al., 2017). MC, pH, TC, and CN ratio are soil environmental factors that can influence the relative abundance of Stramenopiles, which was also the most prevalent protist taxonomic subcommunity observed in our study (Xiong et al., 2018).

Together, these results suggest that soil environmental factors that differ in different soil habitats control soil microbial activity. We showed that environmental variables and community composition were correlated with changes in the dominant phyla of fungi and protozoa across all habitats, which was consistent with the trends in Proteobacteria. The correlation between microbial communities (such as bacteria, fungi, and protists) and environmental parameters (such as MC, pH, TP, and CN ratio) is a critical aspect of understanding the dynamics of soil ecosystems. Our research findings, showing strong and weak correlations between the dominant phyla and the above parameters, provide valuable insights into these relationships. In contrast to typical domains, many studies on bacteria have found that the relative abundance of Proteobacteria decreased from topsoil to subsoil, possibly due to a lack of nutrients (Egidi et al., 2019; Li et al., 2022). In contrast to earlier studies exploring various ecosystems such as fallow fields (Ko et al., 2017), natural forests (Ma et al., 2017), grasslands (Prober et al., 2015), global soil fungal communities, and bacteria land‐use changes from forests to farmland (Egidi et al., 2019; Kim et al., 2021; Li et al., 2022), our results present distinct outcomes for the soil profiles in the specific context of urban soils. These disparities in findings underscore the unique characteristics and dynamics of the soil microbial communities within our study area. A study conducted by Xu et al. (2014) also reported that Proteobacteria, Planctomycetota, and Chloroflexi were among the most dominant phyla in 16 representative Chinese urban parks. In addition, proteobacteria were also typically found in freshwater ecosystems (Yang et al., 2022), where dominant phyla appeared to be essential to the functioning of wetland ecosystems. Proteobacteria have important roles in organic decomposition and recycling and are a group of bacteria that can live in an environment with nutrition and sunlight (Yang et al., 2022). The life history strategy can also explain the dominance of taxonomic distribution among soil habitats. Proteobacteria taxa are traditionally fast‐growing and prefer a soil environment with abundant nutrients (Li et al., 2022).

The majority of the three domains examined in this study showed different responses to TC, CN ratio, MC, and TN, indicating weaker effects of these soil characteristics on shaping the communities of bacteria, fungi, and protist domains. Noticeably, we showed that microbial domains tended to be more similar in sites associated with urbanization than those in farmland and forest sites (Goss‐Souza et al., 2017). Whereas pH was potentially associated with samples in urban areas, nutrients (CN ratio, TC, and TN) were related to soil samples collected from forest ecosystems. These observations correspond with studies on fungal community composition in urban park soils in Shanghai (Zhang et al., 2021) and in Southwestern urban soils of the United States of America (Chen et al., 2021). Although many environmental variables were found to have a significant impact on bacteria, fungi, and protist domains, our variation partitioning analysis results revealed that environmental and spatial factors only play a minor role in determining bacteria, fungi, and protist domains in all subcommunities (CRAT‐CAT, CRT, and rare taxa). This was demonstrated by the small percentage of domain variation that could be explained by these two factors.

The unexplained variance of >90% refers to the cumulative unexplained variance across all domains (bacteria, fungi, and protists) and their respective subcommunities (CRAT and CRT). It represents the combined portion of variation in the data that cannot be accounted for by the spatial and environmental factors considered in the study. In contrast, previous studies using variation partitioning analysis across various habitats and sampling areas (Chen et al., 2017) have shown that significant amounts of unexplained rare bacterioplankton and microeukaryotic community variation can be attributed to the exclusion of certain influential characteristics in the analysis. For instance, Chen et al. (2017) considered variables such as water temperature, dissolved oxygen levels, and nutrient concentrations in their analysis, while (Lindstrom & Langenheder, 2012) incorporated factors like sediment composition, light availability, and organic matter content. The absence of such variables in our study might explain the substantial variance that remains unexplained by spatial and environmental factors. Second, the variation partitioning analysis tends to undervalue the contribution explained by environmental factors, which may also be a possible cause of the low influence of deterministic or selective processes on the variance of the micro eukaryotic and procaryotic communities (Gilbert & Bennett, 2010). Indeed, variation partitioning analysis is frequently used in ecological research to assess the relative effects of environmental selection and spatial impacts on the structure of microbial communities, several studies have used it to understand the effects of ecological processes (Gilbert & Bennett, 2010). Variation partitioning analysis should also be used as an exploratory tool in conjunction with other approaches to develop hypotheses and ascertain the relative significance of environmental and spatial variables. As a result, caution should be used when using variation partitioning analysis to investigate community variation.

### 4.3 Ecological processes driving microbial community assembly

Microbial community ecology is moving from the explanation of patterns in the composition of microorganisms to the explanation of the mechanisms underlying community assembly (Sloan et al., 2006). In urban ecosystems, nutrient availability, MC, water temperature, and pH are known strong factors that influence communities of bacteria, fungi, and protists (Nyirabuhoro et al., 2020). We anticipated that similar environmental factors, across microbial domains, would have strong effects in structuring our microbial subcommunities. Surprisingly, we found that all subcommunities correlated significantly with various environmental factors, demonstrating antagonistic effects of environmental variables on bacteria, fungi, and protists. We suspect that unmeasured environmental factors (e.g., ambient temperature, food, pollutants, population density, and light), which require further investigation, had important effects even though there was no specific environmental variable underlying the dynamics of CRT in any domain. Recent studies have shown environmental filtering as an example of deterministic process and dispersal limitations as an example of a stochastic process that are likely to affect temporal changes in microbial community structure mechanism (Rosindell et al., 2011; Sloan et al., 2006).

Our study contributes to the understanding of assembly mechanisms in an urban soil ecosystem by comparing the dynamics of bacteria, fungi, and protists, including their subcommunities of both rare and abundant taxa. We show that the values of the neutral community model parameter and *R* ^2^ were slightly higher in bacteria‐CRT than that of CRAT + CAT. A good fit to the neutral community model for this subcommunity indicates that these taxa were more strongly influenced by stochastic processes. In contrast, deterministic processes were more important in structuring the rare subcommunity of bacteria, all fungi subcommunities, and protists‐CRAT‐CAT. Among all taxonomic subcommunities studied, the bacteria‐CRT domain displayed the highest degree of stochasticity, with a proportion of 75% exceeding that of all other domains. In addition, the value of metacommunity size multiplied by immigration in bacteria was much higher for the CRT than in other subcommunities, implying that the dispersal of microbial taxa in the bacteria‐CRT was less limited than that of protists‐CRT. Our variation partitioning analysis results demonstrated that environmental factors had little impact on the distribution of bacteria, fungi, and protists even though CRT fit the neutral model. This demonstrates that without the use of complementary models, the neutral model cannot directly predict the mechanism shaping microorganisms (Gotelli, 2006).

Furthermore, when it came to the community immigration rate, the m values in protists‐CRT were higher than those in fungi. The more homogeneous the habitat, the better the results. There is still a disagreement regarding how deterministic and stochastic mechanisms affect microbial domains. It was suggested by Campbell et al. (2011) that deterministic changes should be connected to environmental causes and interactions between microbial taxa given that both deterministic and stochastic processes have an impact on the dynamics of the CRT community (Nyirabuhoro et al., 2021). A partial conclusion is that stochastic processes strongly influenced both bacterial (CRAT‐CAT, CRT) and protist (CRT) assemblages. Deterministic processes had significant impacts on how the protists (CRAT‐CAT) and fungi (CRAT‐CAT, CRT, and rare taxa) behaved. Though both deterministic and stochastic ecological processes play significant roles in shaping the microbial domain, bacteria‐CRT domains that are affected by stochastic processes may be better suited to predicting community change (Nyirabuhoro et al., 2020; Shade et al., 2014). Overall, our results indicate that CRT can be a seed bank for the bacterial and protist domains in the urban soil ecosystem, and they might play a crucial role in maintaining the stability of the most dominant phyla (bacteria; proteobacteria, and protists; stramenopiles) in urban soil ecosystems under a changing environment.

Our results have a major limitation of the subcommunities obtained from environmental DNA, which barely reflects the functional communities of organisms. Metabarcoding approaches fail to expose dynamic attributes, including the spatiotemporal activity of the entire community and the influence of the environment on such activities. Further research amalgamating supplementary information in metagenomic analyses would be beneficial by furnishing enhanced perspectives of microbial profiles.

## 5 CONCLUSION

This study highlights the significant importance of examining transient taxa to get a more complete view of the microbial diversity in urban soil systems. CRT were the most dominant subcommunity among four studied subcommunities namely CRAT, CAT, CRT, and rare taxa. These subcommunities did not show similar patterns, suggesting a large difference in their environmental responses. Stochastic processes had a strong effect on bacteria‐CRAT‐CAT, bacteria‐CRT, and protists‐CRT, and were more pronounced within the bacteria‐CRT subcommunities. In contrast, deterministic processes mainly governed fungi across all subcommunities. Overall, our findings show that CRT in the urban soil ecosystem could potentially serve as a seed bank for bacterial and protistan domains. However, further research is necessary to confirm this hypothesis and provide definitive evidence.

## AUTHOR CONTRIBUTIONS

**Alexis Kayiranga:** Data curation (equal); formal analysis (equal); investigation (equal); methodology (equal); visualization (lead); writing – original draft (lead); writing – review and editing (lead). **Alain Isabwe:** Formal analysis (equal); investigation (equal); methodology (equal); visualization (equal); writing – original draft (equal); writing – review and editing (equal). **Haifeng Yao:** Conceptualization (supporting); data curation (supporting); project administration (lead); resources (equal); validation (supporting); visualization (supporting); writing – original draft (supporting); writing – review and editing (supporting). **Huayuan Shangguan:** Data curation (supporting); project administration (supporting); validation (supporting); writing – original draft (supporting); writing – review and editing (supporting). **Justin Louis Kafana Coulibaly:** Formal analysis (supporting); investigation (supporting); writing – original draft (supporting); writing – review and editing (supporting). **Martin Breed:** Writing – original draft (supporting); writing – review and editing (supporting). **Xin Sun:** Conceptualization (lead); funding acquisition (lead); resources (lead); supervision (lead); writing – original draft (equal); writing – review and editing (equal).

## CONFLICT OF INTEREST STATEMENT

The authors affirm that there are no competing interests present in this work.

## Supporting information


Figure S1:

## Data Availability

Raw sequences data are available from NCBI under the accession number PRJNA1001256. R Scripts used in data analysis and visualizations are available at https://github.com/isalain/soil‐microbial‐subcommunities.
