# Supplementary material for: Distribution patterns of soil bacteria, fungi, and protists emerge from distinct assembly processes across subcommunities
Source: Ecol Evol. 2024 Jul 10;14(7):e11672. doi: 10.1002/ece3.11672 (PMC11236429; doi:10.1002/ece3.11672)
Supplement: Supplementary file 1 — Figure S1: [file ECE3-14-e11672-s001.docx]

SUPPLEMENTARY MATERIAL OF THE ARTICLE:

**Distribution patterns of soil bacteria, fungi, and protists emerge from distinct assembly processes across subcommunities**

Alexis Kayiranga^a,b,c^, Alain Isabwe^a^, Haifeng Yao^a,b,c^, Huayuan Shangguan^a,b,c^, Justin Louis Kafana Coulibaly^a,b,c^, Martin Breed^d^, Xin Sun^a,b,c^*

^a^ Key Laboratory of Urban Environment and Health, Institute of Urban Environment, Chinese Academy of Sciences, 1799 Jimei Road, Xiamen 361021, China.

^b^ University of Chinese Academy of Sciences, Beijing 100049, China.

^c^ Zhejiang Key Laboratory of Urban Environmental Processes and Pollution Control, CAS Haixi Industrial Technology Innovation Center in Beilun, Ningbo 315830, China.

^d^ College of Science and Engineering, Flinders University, Bedford Park, SA 5042, Australia.

*Corresponding author: [xsun@iue.ac.cn](mailto:xsun@iue.ac.cn)

THIS SUPPLEMENTARY MATERIAL CONTAINS:

- Figure S1: Rarefaction curves
- Figure S2: Phylum-level taxonomic composition of bacterial subcommunities
- Figure S3: Phylum-level taxonomic composition of fungal subcommunities
- Figure S4: Phylum-level taxonomic composition of protistan subcommunities
- Table S1: The relative number of OTUs and reads
- Table S2: Extended results of the variation partitioning analysis


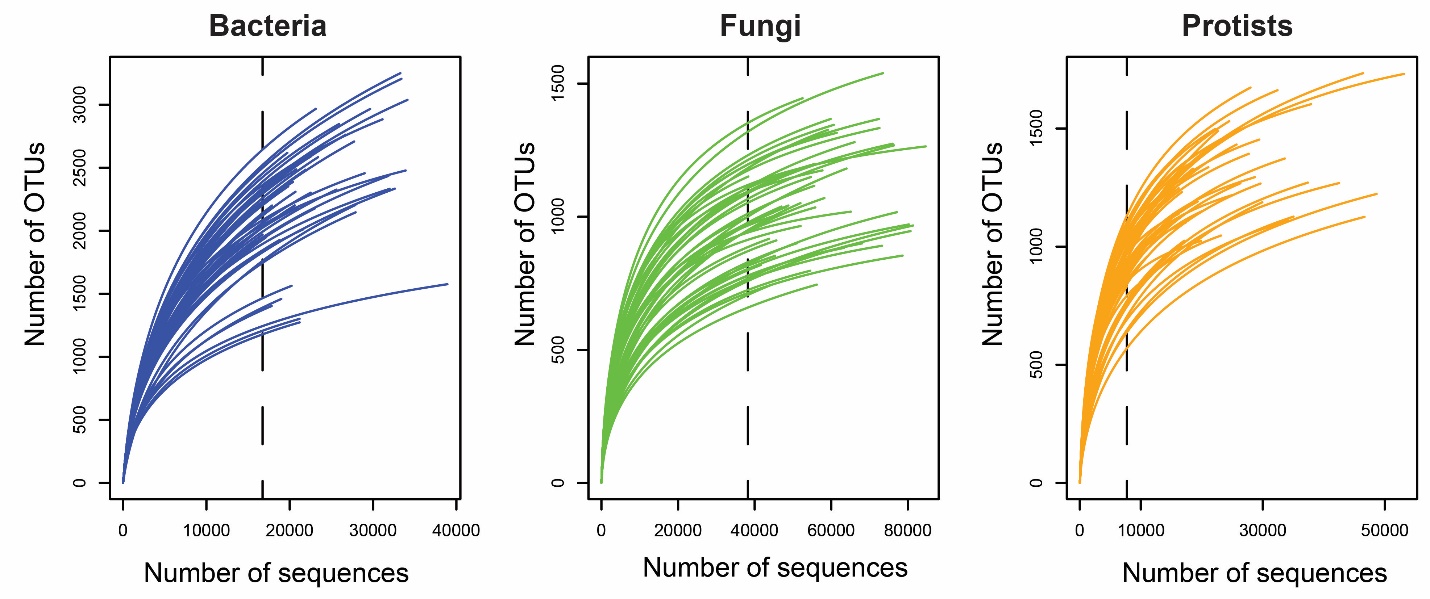


**Fig S1.** Rarefaction curves showing the observed number of OTUs in soil samples collected from 46 sites along an urbanization gradient in Ningbo City, China. The rarefaction cut-off (dashed vertical line) was set when the curves tended to plateau as only the rarest taxa remained to be sampled. The argment ‘step’ was set to 200 to samples the curve at intervals of 200 OTUs. A total of 200, 377, and 856 OTUs were removed because they are no longer present in any sample after random subsampling, respectively for bacteria, fungi and protists.


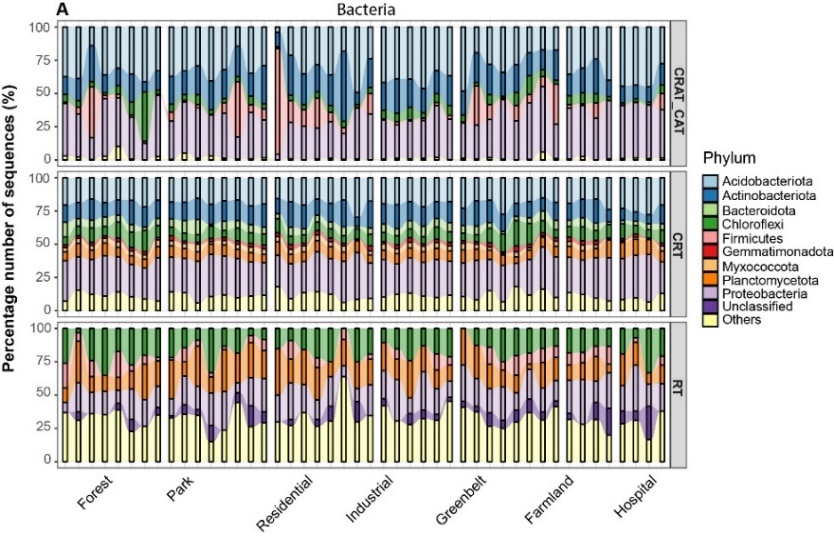


**Fig S2**. Phylum-level taxonomic composition of bacterial subcommunities. Taxa with a relative abundance < 1 % in a sample were assigned to “Others”.


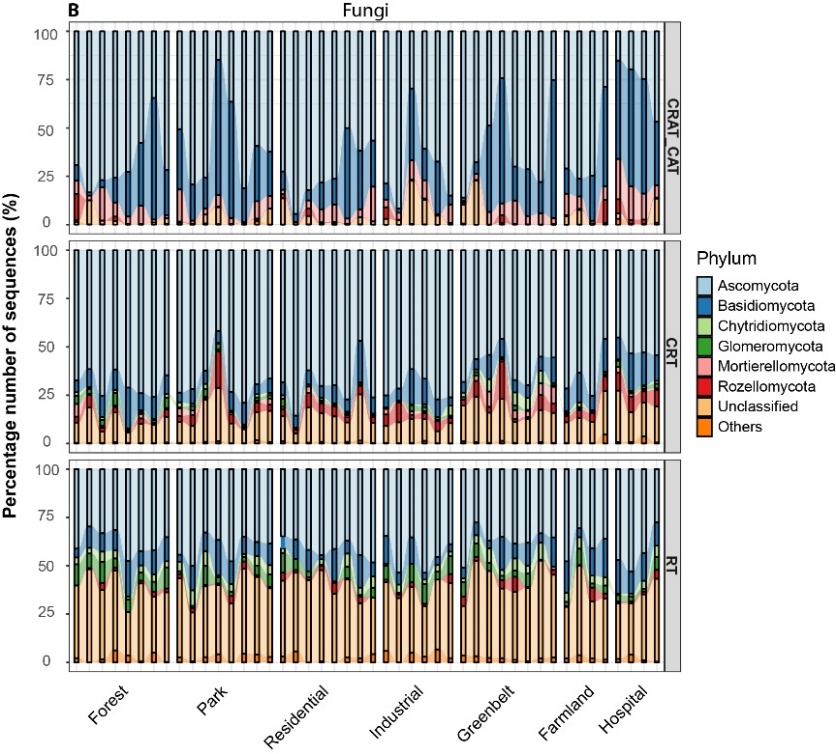


**Fig S3** Phylum-level taxonomic composition of fungi subcommunities. Taxa with a relative abundance < 1 % in a sample were assigned to “Others”.


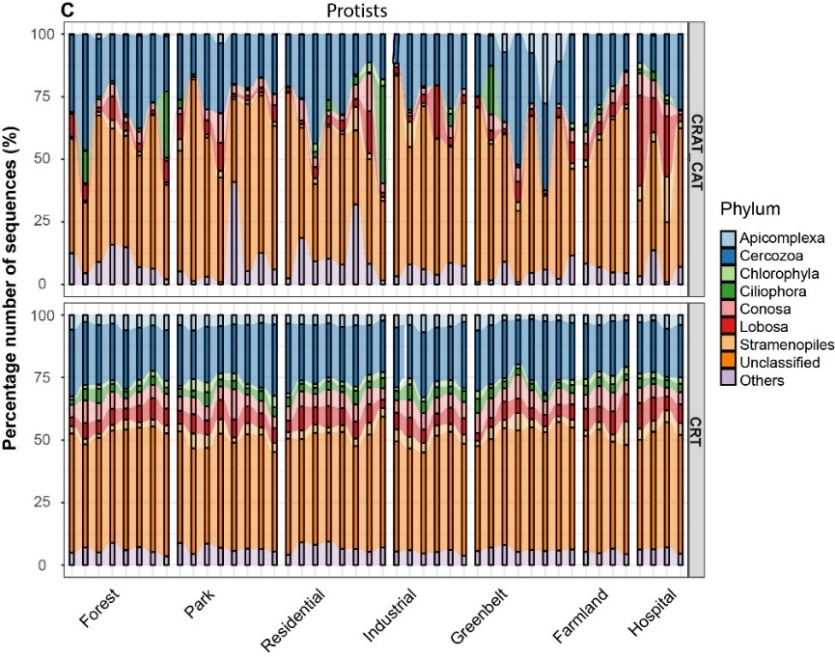


**Fig S4**. Phylum-level taxonomic composition of protist subcommunities. Taxa with a relative abundance < 1 % in a sample were assigned to “Others”.

**Table S1.** Absolute number of reads associated with different subcommunities of bacteria, fungi, and protists

| Community | Subcommunity | Absolute number of reads |
| --- | --- | --- |
| Bacteria | RT | 1411 |
|  | CAT | 23972 |
|  | CRAT | 171542 |
|  | CRT | 513654 |
|  | MT | 18212 |
|  |  |  |
| Fungi | RT | 8796 |
|  | CAT | 26055 |
|  | CRAT | 1187858 |
|  | CRT | 435684 |
|  | MT | 0 |
|  |  |  |
| Protists | RT | 3255 |
|  | CAT | 24916 |
|  | CRAT | 177556 |
|  | CRT | 149623 |
|  | MT | 0 |

**Table S2.** Extended results of the variation partitioning analysis (VPA) describing factors that affect soil microbial community composition across 7 land use types with all fractions

|  |  | |  |  |  |  |  |  |  |  |  |
| --- | --- | --- | --- | --- | --- | --- | --- | --- | --- | --- | --- |
|  | Bacteria | | | |  | Fungi | | |  | Protists | |
|  | CRAT-CAT | CRT | | RT |  | CRAT-CAT | CRT | RT |  | CRAT-CAT | CRT |
| Spatial | 0.27 | 0.16 | | 0.01 |  | 0.06 | 0.04 | 0.02 |  | 0.06 | 0.06 |
| Env. | 0.46 | 0.36 | | 0.04 |  | 0.14 | 0.10 | 0.02 |  | 0.17 | 0.13 |
| Spatial ∪ Env. | 0.48 | 0.37 | | 0.05 |  | 0.14 | 0.10 | 0.03 |  | 0.18 | 0.14 |
| Spatial\|Env. | 0.02 | 0.01 | | 0.01 |  | 0.00 | 0.00 | 0.01 |  | 0.01 | 0.01 |
| Env. ∩ spatial | 0.25 | 0.15 | | 0.00 |  | 0.06 | 0.04 | 0.01 |  | 0.05 | 0.05 |
| Env.\|spatial | 0.21 | 0.21 | | 0.04 |  | 0.08 | 0.06 | 0.01 |  | 0.12 | 0.08 |
| Residuals | 0.52 | 0.63 | | 0.95 |  | 0.86 | 0.90 | 0.97 |  | 0.82 | 0.86 |
| Forward-selected Env. | pH; CN | pH;CN;TP ;MC | | TP; pH; CN; |  | pH;TP;TC | pH; TP; CN | pH |  | pH; TP; TC | pH;TP;CN MC |
| Forward-selected PCNMs | PCNM1;20;7;13 | PCNM1;20 | | PCNM1; 13 |  | PCNM1;5;20 | PCNM1;20 | PCNM1; 8 ;7 |  | PCNM1 ;20 | PCNM1;5;20;9 |

PCNM were principal coordinates of neighbor matrices vectors with positive spatial autocorrelation, which were used as the proxies for spatial variables n=46

Env. environmental variables.
